# Examining the relationship between non-suicidal self-harm and suicidality within the past 12-months and gaming problems in Norwegian full-time students

**DOI:** 10.1186/s12888-024-05694-3

**Published:** 2024-03-28

**Authors:** Tony Leino, Turi Reiten Finserås, Jens Christoffer Skogen, Ståle Pallesen, Joakim Hellumbråten Kristensen, Rune Aune Mentzoni, Børge Sivertsen

**Affiliations:** 1 Department of Psychosocial Science, University of Bergen, Bergen, 5015 Norway; 2 Norwegian Competence Center for Gambling and Gaming Research, University of Bergen, Bergen, 5015 Norway; 3 Department of Health Promotion, Norwegian Institute of Public Health, Bergen, 5015 Norway; 4 Centre for Evaluation of Public Health Measures, Norwegian Institute of Public Health, Oslo, 0473 Norway; 5 Alcohol and Drug Research Western Norway, Stavanger University Hospital, Stavanger, 4068 Norway; 6 Department of Research and Innovation, Helse Fonna HF, Haugesund, 5525 Norway

**Keywords:** Internet gaming disorder, Young adults, Sex, Engaged gamers, Non-suicidal self-harm, Suicidality

## Abstract

**Background:**

Although gaming problems are associated with poor mental health, few population-based studies have examined its association with self-harm and suicidality. This study investigates the association between gaming problems, non-suicidal self-harm and suicidality within the past year, stratified by sex among Norwegian full-time students.

**Methods:**

Data derived from the Norwegian Students’ Health and Wellbeing Study 2022 (*N* = 59,544). The respondents were categorized into non-gamers, recreational gamers, engaged gamers, problematic gamers, and addicted gamers based on the *Game Addiction Scale for Adolescents*. Log-link binomial regression models, stratified by sex, adjusted for age, were used to estimate the risk ratio of non-suicidal self-harm (ideation and behavior) and suicidal behaviors (ideation and attempt) across different levels of gaming problems.

**Results:**

Among females, the risk of non-suicidal self-harm and suicidal ideation increased from non-gamer to problem gamer, with no differences between problem and addicted gamers. Among males, the risk of non-suicidal self-harm increased from non-gamers to engaged gamers, but no differences were observed between engaged, problematic, and addicted gamers. No sex × gaming category interaction was observed for suicide attempts. Engaged and addicted gamers had higher risks of suicide attempt than non-gamers and recreational gamers.

**Conclusions:**

Gaming problems are associated with increased risk of non-suicidal self-harm and suicidal ideation among females. Among males, no differences were observed between engaged, problem and addicted gamers. The results highlight sex when studying health related outcomes and their association to level of gaming problems. Longitudinal studies are warranted to uncover the temporal mechanisms between IGD, non-suicidal self-harm and suicidality.

**Supplementary Information:**

The online version contains supplementary material available at 10.1186/s12888-024-05694-3.

## Introduction

Video gaming has become one of the most popular leisure activities of our time. In Norway, the context of this investigation, a recent study of the general population found that 46% of the respondents (16–74 years) had played video games (computer and/or video game consoles) during the last 6 months [1]. Similar to other studies, a larger proportion of males (53%) had played compared to females (38%), and participation decreased with age [1]. Although most gamers will not experience gaming as harmful, some develop problems with controlling the activity. While the exact pathway leading to gaming problems cannot clearly be delineated, it often seems to start with an increased preoccupation, followed by diminished interest in other activities, frustration when gameplay is limited, neglect of responsibilities (school/work), and disregard for interpersonal relationships, culminating in an inability to control the activity [2]. Such problems can become so severe that the gamer might fulfill the diagnostic criteria for gaming disorder (GD) or internet gaming disorder (IGD) found in current psychiatric nosology. Gaming disorder (GD) was included as a diagnosis in the eleventh edition of *International Classification of Diseases* (ICD-11) [3], whereas Internet Gaming Disorder (IGD) was included as a condition for further study in the fifth edition of the *Diagnostic and Statistical Manual of Mental Disorders* (DSM-5) [4]. In addition to these terms, “addicted gaming” is frequently employed in research, especially when studies categorize participants into different gaming groups [5, 6]. These terms are often used interchangeably in the literature. In this paper problem gaming refers to clinical and/or subclinical problems related to gaming that implicate impaired control and negative consequences but are not necessarily serious enough to fulfill diagnostic criteria for IGD. IGD, GD, and gaming addiction (referring to “addicted gamers” when describing affected individuals) are treated equally and refer to impaired control and negative consequences in line with diagnostic criteria for IGD.

IGD implies a persistent and recurrent involvement with video games that causes distress and impairs daily living and functioning [7]. A recent meta-analysis estimated the global prevalence of IGD to be 3.3%. However, when restricting the analysis to representative samples, the pooled estimate dropped to 2.4% [8]. In Norway, the prevalence of game addiction among individuals aged 16–74 years was 0.7% in 2022, while 5.0% were categorized as problem gamers [1] IGD is more prevalent among males than females and is more common among adolescents and young adults as compared to adults [9, 10]. For example, the Norwegian study among 16–74 year olds showed that 8.0% of males and 3.2% of females could be categorized as either problem gamers or gaming addicts whereas the corresponding figures among individuals aged 16–25 years was 23.7% and 9.6%, respectively [1]. This sex difference can be (partly) explained by the higher prevalence of males playing video games [11], which is influenced by the historical targeting of video games towards males [12], while females have been more likely to be discouraged from participating in gaming and due to an increased likelihood of experiencing negative events when participating in gameplay (e.g., being harassed and bullied) [13].

IGD is associated with poor mental health, sleep problems, psychopathology, and other social- and health-related outcomes [9, 14–16]. A comprehensive review [14] reported moderate to large effect sizes between IGD and depression, ADHD, and social phobia. Findings show that suicidality is prevalent among individuals with gaming problems. A recent literature review of 12 studies [17] found a positive relationship between problem gaming and suicidal ideation and/or attempts. Similarly, in a large-scale purposive sampling survey (*N* ~ 3400) among individuals aged 11–35 years [18], it was found that individuals with IGD had greater odds of suicidal ideation (OR = 2.2), self-harm (OR = 2.3), and suicide attempts (OR = 2.7), compared to individuals without IGD. Interestingly, age-stratified analyses found a positive relationship between IGD, self-harm and suicidal behaviors among those aged 11–17 years, but not among those aged 18–35 years. Furthermore, Ohayon et al. [19] found that individuals with IGD showed a higher rate of suicidal ideation (16.9% vs. 6.6%), suicide attempts (9.7% vs. 3.3%), major depressive disorder (9.7% vs. 3.0%), and social anxiety disorder (24.8% vs. 8.5%) compared to individuals without IGD among US university students (18 + years).

Few studies have investigated the relationship between IGD and non-suicidal self-harm. Evren et al. [20] found a positive association between IGD and non-suicidal self-harm among university students, even after controlling for depression, anxiety, and neuroticism. Still, their results also suggest that the relationship between IGD and non-suicidal self-harm may be confounded by psychological impairment. Although psychological distress might be an essential factor for non-suicidal self-harm and suicidal behaviors, the temporal, and causal factor between psychological distress and IGD remains unelucidated [21].

Hufford [22] suggests that distal factors, such as psychopathology and negative life events create a stable potential for suicidal behavior, while proximal factors, such as acute psychological distress, hopelessness, and loneliness, are linked to the timing of the event. In line with this framework, gaming problems could increase the risk of non-suicidal self-harm and/or suicidality by acting as either a distal factor, creating a stable potential, and/or a proximal triggering factor by exacerbating the total amount of distress caused by other distal conditions. However, the self-medication hypothesis [23, 24] of addictive disorders postulates that some individuals experiencing suicidality may develop gaming problems because they use gaming to cope with, or escape from, the distress associated with their suicidality. In line with this perspective, gaming may decrease the likelihood of suicidal behaviors. Moreover, affect regulation, including distraction, relief of negative emotions, and stress management, appear to be the primary motivation behind non-suicidal self-harm behavior [25]. Hence, this framework suggests that gaming could decrease the likelihood of non-suicidal self-harm (ideation and behavior) and suicidality. However, such a coping strategy can be suboptimal as it could lead to gaming problems [26]. However, due to the lack of longitudinal designs, the causal mechanism between IGD, self-harm, and suicidality is not known [17]. This is similar to other technological addictions, such as internet addiction where some findings show that internet addiction predicts self-harm/suicidal behavior [27] while others do not [28].

Both theoretical suggestions and empirical findings suggest the need to distinguish between engaged and problem/addicted gamers [6, 29–31]. For example, Charlton et al. [6, 29] show that gaming addiction is directly linked to core addiction criteria (release and reinstatement, conflict, withdrawal, and behavioral salience), whereas engagement is linked to peripheral addiction criteria (cognitive salience, tolerance and euphoria). In line with this, a meta-analysis concludes that failing to make such distinction leads to spuriously high prevalence estimates of gaming addiction as engaged gamers often are classified as addicted [32].

A previous study from the Norwegian Students’ Health and Wellbeing Study (*N* = 59 544, 18 years or older) examining mental distress and life satisfaction among different categories of gamers (non-gamer, recreational gamer, engaged gamer, problem gamer, addicted gamer) found that the proportion reporting mental distress and poor life satisfaction increased with levels of gaming problems [33]. Of particular importance, the results showed large sex differences, where a larger proportion of females, compared to males, reported mental distress across all gaming categories except among the game addicts. In terms of low life satisfaction, this was reported by a larger proportion of females, compared to males among recreational and engaged gamers. Mental distress is positively linked to non-suicidal self-harm and suicidal behaviors and females report higher levels of mental distress in general. *Ceteris paribus*, this might suggest that females have a higher risk than males of expressing non-suicidal self-harm and suicidal behaviors as gaming participation and/or gaming problems symptoms increase.

Against this backdrop, the aim of the present study was to investigate the risk of non-suicidal self-harm and suicidal behaviors among problem and addicted gamers compared to other types of gamers, by sex in a nationally representative study of college and university students in Norway.

## Materials and methods

### Participants

The current study used data from the Students’ Health and Wellbeing Study 2022 (SHoT2022), a large national survey of students enrolled in higher education in Norway. All full-time college and university students in Norway aged 18 or older were invited to participate in the study. Out of a total of 169 572 eligible students, 59 544 completed the online questionnaire, resulting in a total response rate of 35.1%. Among these, 66.5% were females and the mean age was 26.1 years (SD = 7.3).

### Instruments

Data about the participants’ age and sex were extracted from their 11-digit Norwegian national social security number.

The dependent variables were non-suicidal self-harm ideation, non-suicidal self-harm behavior, suicidal ideation, and suicide attempt.

History of non-suicidal self-harm ideation was examined with one item adapted from the Child and Adolescent Self-Harm study [34]:

“Have you ever seriously thought about trying to deliberately harm yourself but not with the intention of killing yourself, but not actually done so?”.

History of non-suicidal self-harm behavior, suicide ideation, and suicide attempt were assessed by items from the Adult Psychiatric Morbidity Survey [35] using one-item questions for each type of behavior. The specific one-item questions were:

“Have you ever deliberately harmed yourself in any way but not with the intention of killing yourself?”,

“Have you ever seriously thought of taking your life, but not actually attempted to do so?”, and

“Have you ever made an attempt to take your life, by taking an overdose of tablets or in some other way?”

If respondents answered yes to any item, the timing of the most recent episode was assessed using the following response options: ‘*Last week*’, ‘*Past year*’, *More than a year ago, but after I started studying at the university*’, and ‘*Before I started studying at university*’. In the present study, we investigated those with episodes having occurred within the past 12 months (the two first response options).

The independent variable was based on categories derived from the scores on the *Game Addiction Scale for Adolescents* (GASA). GASA is a 7-item scale measuring video game addiction on a five-point Likert scale (1 = never, 2 = almost never, 3 = sometimes, 4 = often, 5 = very often) [36]. In line with the categorization suggested by Brunborg et al. [5] gamers were divided into four groups: 1) recreational gamers, 2) engaged gamers, 3) problem gamers, and 4) addicted gamers. *Addicted gamers* scored at least 3 (“Sometimes”) on all of the four core criteria (relapse, withdrawal, conflict, and problems). *Problem gamers* had a score of at least 3 (“Sometimes”) on two or three of the four core criteria. *Engaged gamers* had a score of 3 (”Sometimes”) on the three peripheral criteria (salience, tolerance, and mood modification) and scored 3 (“sometimes”) on maximum one of the core criteria. *Recreational gamers* were those who confirmed partaking in gaming through the question “*Have you played video games in the last six months?*” but who did not meet the criteria for neither engaged, problem, nor addicted gamer. The remaining study participants were categorized as non-gamers. A similar categorization of gamers has been used in previous studies [33, 37, 38]. Cronbach’s Alpha for GASA was 0.84, reflecting good internal consistency.

### Procedure

All college and university students in Norway, aged 18 or older, were invited via email and SMS, and included both students studying in Norway and Norwegian students studying abroad. SHoT2022 was distributed electronically through a web-based platform and was conducted between February 8 and April 19, 2022. Awareness was boosted via email, SMS, and outreach by student welfare organizations and schools.

### Statistical analysis

The sample characteristics across gaming categories were calculated in terms of mean and standard deviation (*SD*) for continuous variables and in terms of number of observations and proportions for categorical variables. Overall and sex-stratified log-binomial regression models, adjusted for age, were used to estimate the risk ratio (RR) of non-suicidal ideation and behavior, suicidal ideation and suicide attempt last 12 months as dependent variables across gaming categories. A test for sex moderation was conducted by comparing a model with and without the interaction term *level of problem gaming* × *sex*, using likelihood ratio tests. If sex moderation was significant, a sex-stratified model was estimated. If the sex moderation was not significant, an overall model was estimated.

Pairwise comparisons were conducted using Wald tests based on the parameters of the fitted regression models across gaming categories for each sex, and across sex for each gaming category. STATAs *Seemingly unrelated estimation* (SUEST) command was used to test if the estimates of self-harm and suicidal behaviors were similar for the fitted models for females and males at the same levels of problem gaming. Data handling, descriptive statistics and analyses were conducted in Stata version 15.

### Ethical considerations

Informed consent was obtained electronically from all subjects involved in the study after they had received detailed information about the study.

The study was conducted in accordance with the Declaration of Helsinki. The SHoT2022 study was approved by the *Regional Committee for Medical and Health Research Ethics in Western Norway* (no. 2022/326437).

The reporting in the present paper follows the STROBE guidelines [39].

## Results

Table 1 shows that the majority of females were non-gamers whereas the majority of males were recreational gamers. Comparing the row proportion between male and female (highest proportion value vs. lowest) shows that females were 2.5 times more likely to be non-gamers than males. Males were 2 times more likely to be recreational gamers, 4 times more likely to be engaged or problem gamer, and 6 times more likely to be addicted gamers compared to females. At face-value, non-gamers were more heterogeneous compared to those involved in gaming (recreational to addicted gamers). Non-suicidal self-harm ideation and behavior, suicide ideation rose in parallel with increase in problem gaming category. The proportion of suicide attempt showed a general increase from non- to addicted gamers, except for problem gamers, who had a lower proportion of non-suicidal self-harm ideation and behavior and suicidal ideation compared to engaged gamers.

**Table 1:** Descriptive statistics and proportion of non-suicidal self-harm and suicidal behaviors by gaming category

Characteristics	Non-gamer,	Recreational gamer,	Engaged gamer,	Problem gamer,	Addicted gamer,
	*N* = 32 878 (55.7%)	*N* = 22 058 (37.4%)	*N* = 1 849 (3.3%)	*N* = 1 970 (3.3%)	*N* = 297 (0.5%)
**Sex**					
Female	27 432 (69.7%)	10 563 (26.9%)	612 (1.6%)	78 (1.7%)	78 (0.2%)
Male	5 446 (27.3%)	11 495 (58.3%)	1 237 (6.3%)	1 310 (6.7%)	219 (1.1%)
**Age**					
M (*SD*)	26.7 (8.2)	25.4 (6.0)	24.5 (4.2)	25.1 (5.3)	25.0 (4.8)
**Proportion of self-harm and suicidal behaviors**					
Non-suicidal self-harm ideation	2 538 (7.7%)	2 285 (10.4%)	267 (14.4%)	313 (15.9%)	57 (19.2%)
Non-suicidal self-harm behavior	1 088 (3.3%)	1 035 (4.7%)	112 (6.1%)	160 (8.1%)	32 (10.8%)
Suicidal ideation	2 251 (6.9%)	1 972 (8.9%)	259 (14.0%)	332 (16.9%)	62 (20.9%)
Suicide attempt	142 (0.4%)	113 (0.5%)	18 (1.0%)	16 (0.8%)	6 (2.0%)

*M* mean, *SD* Standard deviation

Table 2 shows the Spearman’s ranked correlation between self-harm, suicidal behaviors and GASA scores, which is measured on a continuous scale. All correlation coefficients were positive. More specifically, there exist a moderately strong relationship between self-harm ideation, self-harm, and suicide ideation, while the relationship between self-harm and suicide ideation is moderate. Moreover, the relationship between suicide attempt and the other respective self-harm behaviors and suicidal ideation varies from weak to moderately weak. Additionally, all respective self-harm behaviors and suicidal behaviors are very weakly associated with GASA. Note that the weak relationship between GASA and the other variables is likely due to the skewness of the scores of GASA (i.e., few are engaged gamers, and even fewer are problem and addicted gamers, respectively).

**Table 2:** The association between self-harm, suicidal behaviors, and GASA using Spearmann's correlation

	1	2	3	4
1. Self-harm ideation	-			
2. Self-harm	0.458	-		
3. Suicide ideation	0.472	0.312	-	
4. Suicide attempt	0.152	0.215	0.206	-
5. GASA (cont.)	0.065	0.051	0.072	0.013

*All correlations at p < .01*

*N* = 59 544, *GASA* Game addiction scale for adolescents, *cont.* using continuous scale

Across all dependent variables there was a significant level of problem gaming × sex interaction, except for suicide attempt. Hence, all but the analysis for suicide attempt were stratified by sex. There was a general tendency for a stronger association to the dependent variables for females compared to males across recreational-, problem- and addicted gamers in relation to all outcomes, except for suicide attempt. For suicide attempt, there was a tendency of a stronger association for addicted gamers compared to the other gaming categories.

### Non-suicidal self-harm ideation

**Fig. 1:**
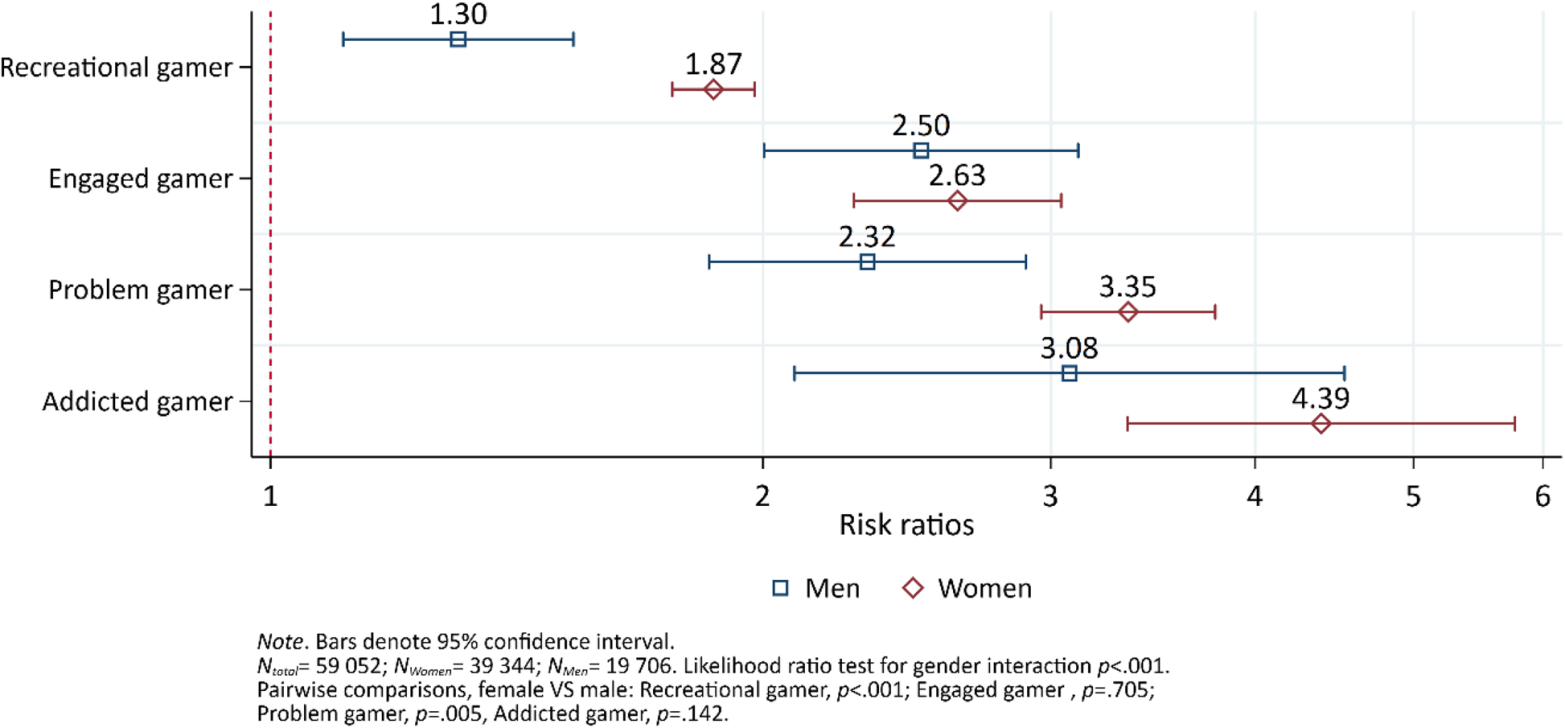
The risk ratio of non-suicidal self-harm ideation by sex and gaming category, adjusted for age. Results from a log-binomial regression model. Non-gamers comprise the reference group indicated by the vertical red dashed line

For females, the risk of non-suicidal self-harm ideation increased by level of problem gaming (Fig. 1). All gaming categories were significantly different from each other (ranging from *p* < 0.001 to *p* < 0.01), except for problem and addicted gamers (*p* = 0.101). A different pattern emerged among males only showing significant differences between recreational gamers and the other gaming categories (ranging from *p* < 0.001 to *p* < 0.01), whereas there were no differences between the other gaming categories (ranging from *p* = 0.205 to *p* = 0.540). Interaction analysis indicated sex differences among recreational and problem gamers where females had a greater risk of non-suicidal self-harm ideation than men among recreational gamers and problem gamers, respectively.

### Non-suicidal self-harm behavior

**Fig. 2:**
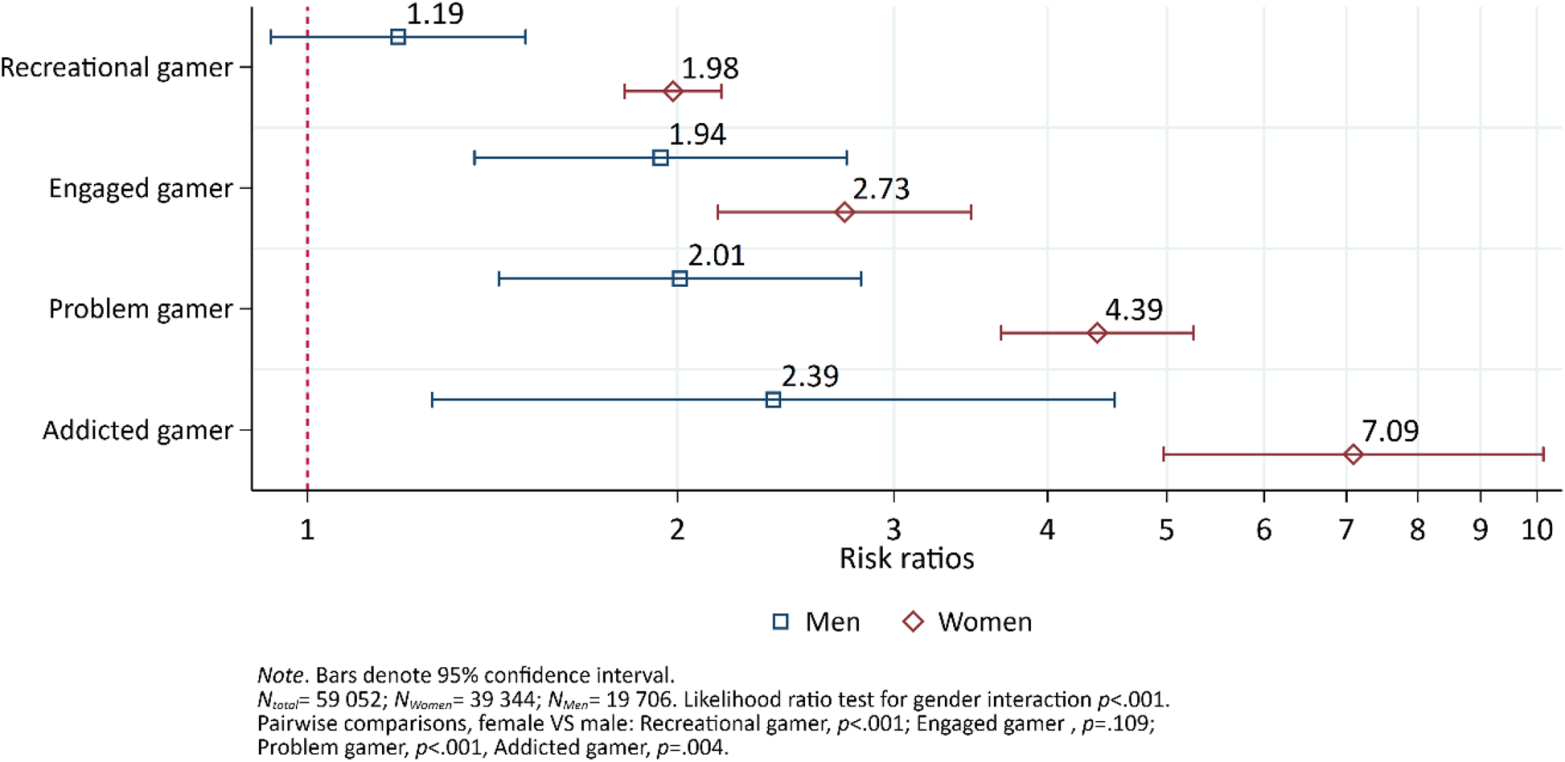
The risk ratio of non-suicidal self-harm behavior by sex and gaming category, adjusted for age. Results from a generalized linear model. Non-gamers comprise the reference group incicated by the vertical red dashed line

Among females, the risk of non-suicidal self-harm behavior increased by level of problem gaming (Fig. 2). All gaming categories were significantly different from each other (ranging from *p* < 0.001 to *p* < 0.05). A different pattern emerged among males. The risk of non-suicidal self-harm behavior was similar between non-games and recreational gamers (*p* = 0.147). Furthermore, the risk of non-suicidal self-harm behavior among recreational gamers was significantly different from the other gaming types (*p* < 0.01), except addicted gamers (*p* = 0.104). There were no other significant differences between the remaining pairs of categories (ranging from *p* = 0.564 to *p* = 0.855). Interaction analysis indicated sex differences, where females had a greater risk non-suicidal self-harm behavior than males among recreational gamers, problem gamers, and addicted gamers, respectively.

### Suicidal ideation

**Fig. 3:**
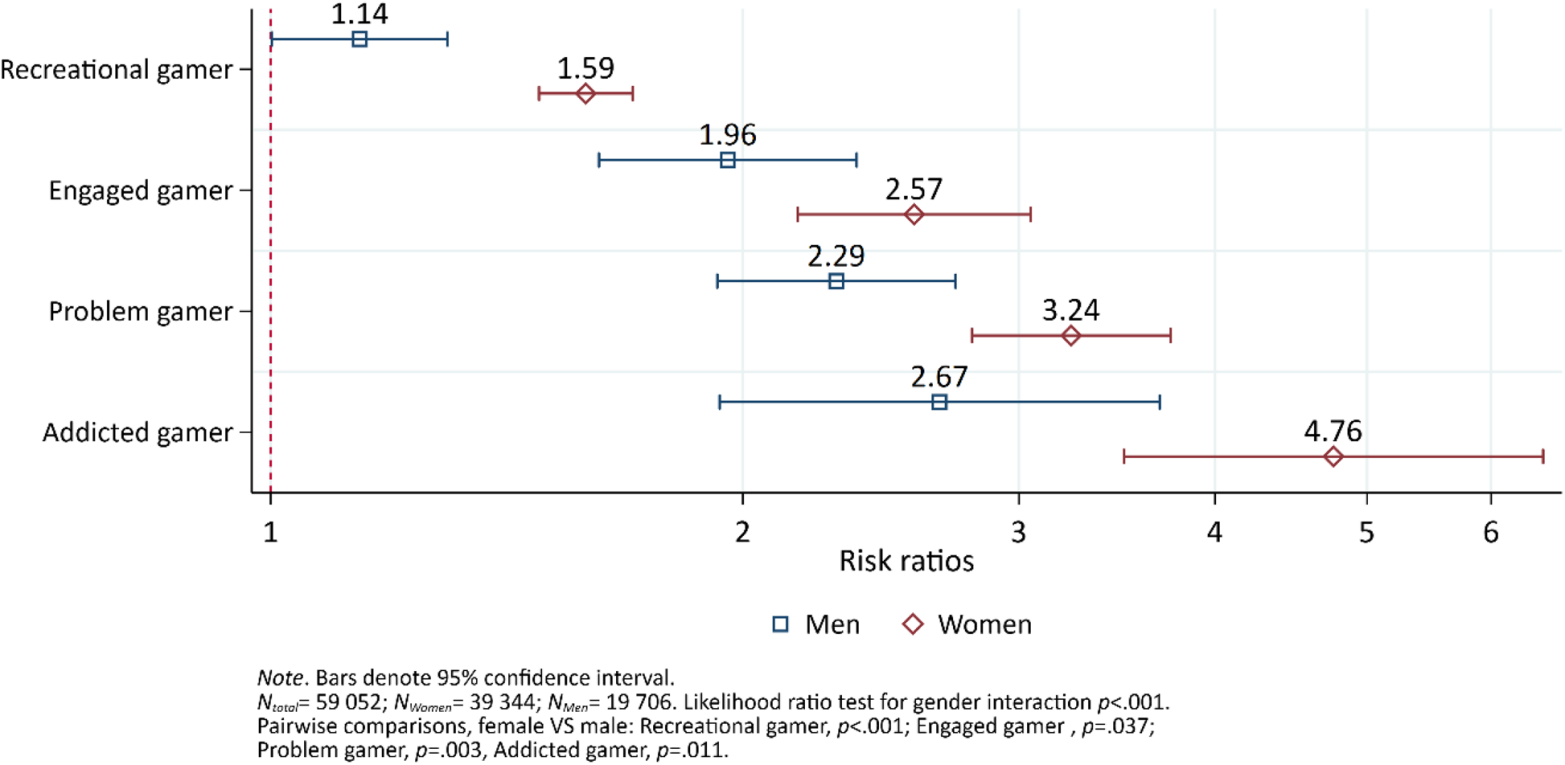
The risk ratio of suicidal ideation by sex and gaming category, adjusted for age. Results from a log-binomial regression model. Non-gamers comprise the reference group indicated by the vertical red dashed line

Among females, the risk of suicidal ideation increased by level of problem gaming (Fig. 3). All gaming categories were significantly different from each other (ranging from *p* < 0.001 to *p* = 0.05). However, among men, the difference in risk of suicidal ideation between non-gamers and recreational gamers was barely significant (*p* = 0.047). Recreational gamers were significantly different from the other gaming categories (*p* < 0.001). There were no significant differences between the other gaming categories (ranging from *p* = 0.073 to *p* = 0.372). Interaction analysis indicated sex differences, where females had a greater risk of suicidal ideation across all gaming categories compared to males.

### Suicide attempt

**Fig. 4:**
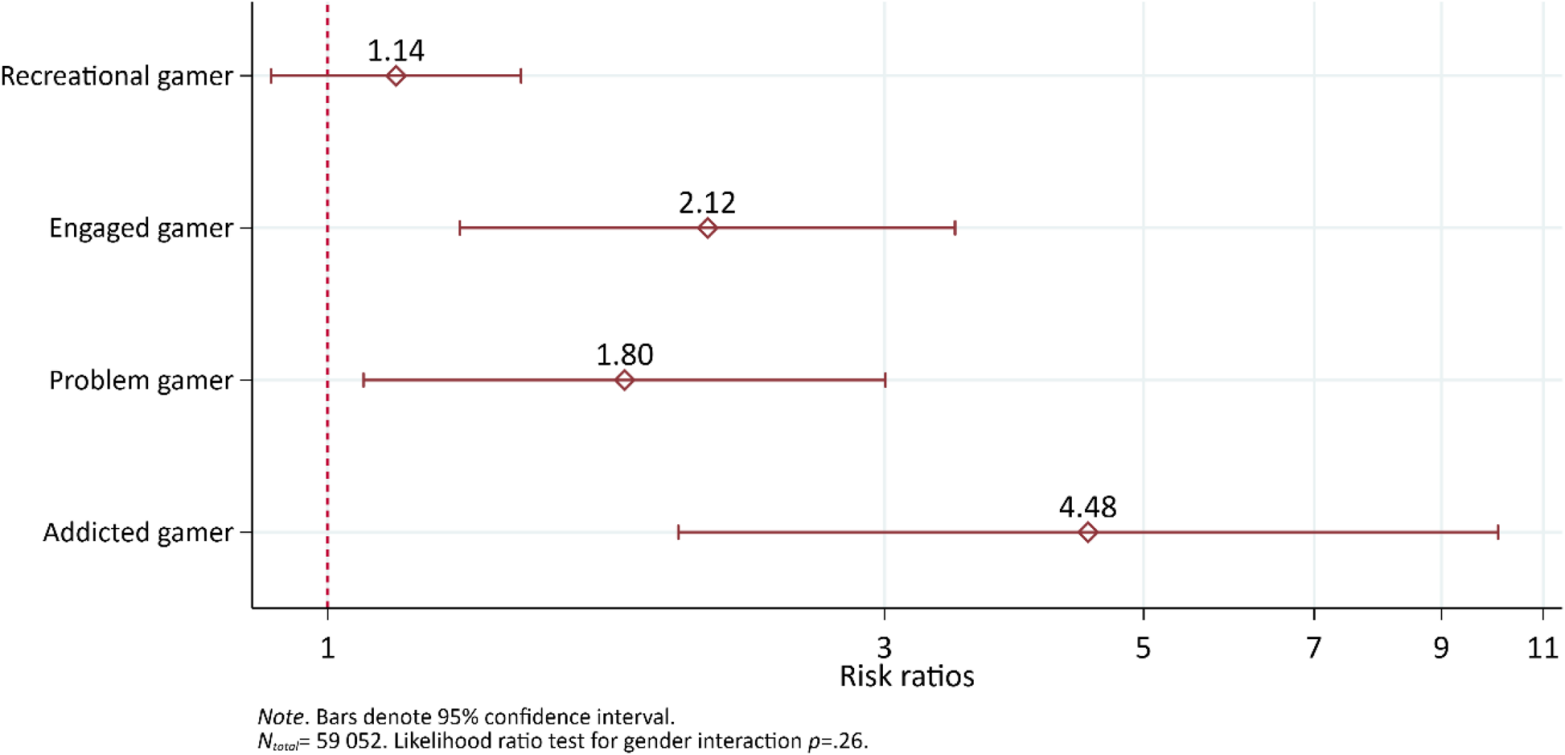
The risk ratio of suicide attempt by gaming category, adjusted for age. Results from a log-binomial regression model. Non-gamers comprise the reference group indicated by the vertical red dashed line

The risk of suicide attempt was greatest among addicted gamers compared to non-gamers (Fig. 4). Similarly, the risk of suicide attempt was greater among engaged- and problem gamers compared to non-gamers, respectively. Recreational gamers were significantly different from engaged gamers (*p* < 0.05) and addicted gamers (*p* < 0.001). There were no other significant differences between the other categories (ranging from *p* = 0.054 to *p* = 0.632).

## Discussion

The aim of the current paper was to investigate the level of problem gaming and its association with non-suicidal self-harm and suicidal behavior, using data from a large-scale national study of full-time students in Norway. The results show a clear sex interaction between non-suicidal self-harm ideation and behavior, and suicidal ideation and level of problem gaming. Among females, the risk of non-suicidal self-harm ideation and behavior, and suicidal ideation increased with greater levels of gaming problems. Among males, the risk of non-suicidal self-harm ideation and behavior, and suicidal ideation was lower among non-gamers and recreational gamers compared to engaged gamers, whereas the other gaming categories were not different from each other. No sex differences were observed in terms of risk of suicide attempt. The results suggest that increasing levels of problem gaming are associated with greater risk of non-suicidal self-harm ideation and behavior, and suicidal ideation among females, but not males. The implications of these findings are discussed based on sex perspective and based on theories of suicide and self-harm.

In line with previous studies, the current findings support an association between non-suicidal self-harm ideation and behavior, and suicidal ideation among engaged and problem gamers among those being 18 years and older [17, 19, 20]. Our findings differ from Junus et al. [18] who found no relationship between self-harm and suicidal behaviors and IGD among participants being 18 years or older. However, there are several differences between the current study and Junus et al. [18] that can account for the varying outcomes. Apart from the different sampling strategies, regional differences, instruments, and sample sizes between the studies, Junus et al. [18] presented adjusted outcomes and did not distinguish between males and females. The failure to identify a significant difference in self-harm and suicidality in their study could be attributed to their adjustment for risk behaviors, psychiatric diagnoses, and internet usage, factors that are likely to mediate the relationship between self-harm and suicidal behavior and gaming (see strengths and limitation section for a further discussion why we did not adjust for mental distress). Furthermore, not separating males and females might also have masked important gender differences in Junus et al. [18].

The present results show generally that female gamers have a greater risk of non-suicidal self-harm ideation and behavior, and suicidal ideation compared to male gamers. Findings from a narrative literature review show that fewer females participate in gaming due to negative expectations and experiences based on gameplay, hyper-sexualization of female attributes, online harassment, and bullying [13]. In addition, females who participate in gaming also report a lack of social support [40]. Consequently, these negative experiences may lead to lower well-being and increased risk of mood disorders, such as depression and anxiety. Indeed, female gamers have a greater risk of reporting mental distress compared to male gamers [33]. Furthermore, whereas males may have a greater risk of developing IGD than females, a greater proportion of females with IGD report loneliness, emotional/mood dysregulation, and affective disorders compared to males with IGD [41]. Hence, female gamers seem to have a greater risk of experiencing both comorbid psychopathology and negative events during gameplay which create a potential for suicidal behavior according to Hufford [22]. Further, female gaming may be regarded as a violation of gender expectations [13], hence part taking in gaming may generally be more socially taxing for females than for males.

In contrast, the self-medication hypothesis of addictive disorders proposes that individuals with suicidal tendencies may develop gaming problems in attempts to cope or escape the distress associated with suicidal behavior [25]. In line with this claim, Melodia et al. [42] argue that an individual may spend more time gaming if the game fulfills an unsatisfied real-life need or alleviates real-life problems, which ultimately may lead to gaming problems. This suggests that some persons who are suffering from non-suicidal self-harm and/or suicidal behaviors may also develop gaming problems as an attempt to regulate these painful experiences and/or urges. Indeed, a study found that the relationship between psychiatric distress and IGD was partly mediated by escape motives and that this effect was stronger for females than males [43]. Similar gender-specific motives have been found for gambling as well [44]. Hence, it could be speculated that the sex differences observed in the present study are likely to reflect that female gamers to a larger extent use gaming to escape from/cope with distress associated with non-suicidal self-harm/suicidal behaviors or other real-life problems than male gamers. Still, future studies with longitudinal designs are needed to uncover the temporal mechanisms between IGD and non-suicidal self-harm and suicidal behaviors.

Although addicted gamers had a greater risk of suicide attempt compared to recreational gamers, no sex differences regarding the risk of suicide attempt by gaming type was observed. This is likely to be due to the low number of addicted gamers (*n* = 297) and the low number of occurrences of suicide attempt in this group (*n* = 6), which constraints our statistical power and limits our ability to conduct a meaningful analysis of the potential interaction between level of problem gaming and sex. Consequently, future studies with a larger group of addicted gamers are needed to uncover this potential relationship.

It is important to consider the distinction between engaged gamers and problem gamers/addicted gamers. While engaged, problem, and addicted gamers may spend the same amount of time gaming, gaming participation among engaged gamers has minimal impact on other aspects of life (e.g., everyday activities and responsibilities) [45]. Indeed, Slack et al. [45] reported that high-frequency gaming becomes problematic when it takes precedence over important daily activities and is used as an escape from real-world problems. Still, engaged gamers had a greater risk of non-suicidal self-harm ideation and behavior, and suicidal ideation compared to recreational gamers for both females and males. This may be due to how engaged gamers are assessed by GASA, a scale developed for measuring gaming problems and not specifically gaming engagement. Consequently, the definition of engaged gamers in the present study included gamers who had a score of three on maximum one of one core criteria of addiction, while recreational gamers could have a score of three on two on the peripheral criteria and one of the core criteria of addiction.

### Strengths and limitations

The response rate in the current study was modest (35.1%). It should be noted that falling response rates in surveys have been quite common in the last decades [46]. Consequently, the results of the study may not be generalizable to other populations than young students in higher education. An analysis of the non-response pattern and its association with sociodemographic variables could have provided a better understanding of respondents and non-respondents. However, information about non-participants was not available in the current study, precluding us from conducting attrition analyses. Still, low response rates are less likely to have an impact when examining relationships between variables compared to descriptive epidemiological endeavours [47]. The current study is also prone to self-report biases, such as social desirability [48], recall bias [49], and common method bias [50].

The classification method for different types of gamers employed in the present study may lead to a higher number of individuals categorized as addicted gamers. Some previous studies have categorized addicted gamers based on the GASA as those endorsing all seven items, resulting in a relatively low proportion of affected individuals [8]. Nevertheless, our objective was to accurately classify the appropriate number of gamers as addicted and avoid under-identifying individuals within this category. A prior study has proposed that classifying gamers into four distinct groups, as in the present study, is a more suitable approach than using more conservative cut-offs and differentiating between fewer groups of gamers [5].

The present study did not account for psychological distress. Accounting for psychological distress suggests a cause-and-effect relationship between psychological distress and IGD (e.g., psychological distress cause IGD). Still, a causal connection between IGD and psychological distress remains uncertain [21]. Furthermore, although psychological distress may be an important confounder in the relationship between IGD and non-suicidal self-harm and suicidality (e.g., 14), the aim of the current paper was to examine this relationship by sex, and not to extricate potentially underlying causal mechanisms.

The current study did not adjust for other potentially important factors that may influence the relationship between self-harm/suicidality and gambling problems, including socioeconomic status, type of educational institution and/or program, and characteristics of the student’s living situation (e.g., living with others/living alone). Consequently, future studies should explore the effect of these factors, and perhaps more importantly, if they differ by sex and how they relate to gaming problems over time. Systematically mapping the factors that influence the relationship between self-harm/suicidality and gaming may result in early identification of at-risk individuals and may as such pave the way for institutional prevention programs.

Although the present study could not examine the relationship between IGD and suicide attempt by sex, a considerable strength of the current study is the large sample size, and the recency of the data collection as well as the use of validated tool to assess both gaming and non-suicidal self-harm suicidality. The large sample allowed for examination of self-harm behaviors and suicidal ideation across different game categories, stratified by sex and with high statistical power.

## Conclusion

The aim of the present study was to examine different typologies of gamers and their association with non-suicidal self-harm and suicidality. The results show that greater levels of problem gaming were associated with increased risk non-suicidal self-harm ideation and behavior, and suicidal ideation among females, but not among males. Studies with longitudinal designs are warranted to uncover the temporal mechanisms between IGD and non-suicidal self-harm and suicidality, as well as investigate the temporal ordering of these two phenomena. In addition, our findings strongly suggest that future gaming studies examining health-related outcomes should be broken down by sex.

### Supplementary Information

**Supplementary Material 1.**

## Data Availability

The datasets presented in this article are not readily available because of privacy regulations from the Norwegian Regional Committees for Medical and Health Research Ethics (REC). Approval from REC (https://www.forskningsetikk.no/en/about-us/our-committees-and-commission/rek/ is a pre-requirement. Guidelines for access to SHoT data are found at: https://www.fhi.no/en/more/access-to-data. Requests to access the datasets should be directed to borge.sivertsen@fhi.no.

## References

[1] Pallesen S, Mentzoni RA, Syvertsen A, Kristensen JH, Erevik EK, Morken AM (2023). Omfang av penge- og dataspillproblemer i Norge 2022.

[2] King DL, Delfabbro PH (2018). Internet gaming disorder: Theory, assessment, treatment, and prevention.

[3] World Health Organization. International statistical classification of diseases and related health problems (11th ed.). WHO. 2018.

[4] American Psychiatric Association. Diagnostic and statistical manual of mental disorders (5th ed.). American Psychiatric Publishing. 2013.

[5] Brunborg GS, Hanss D, Mentzoni RA, Pallesen S (2015). Core and peripheral criteria of video game addiction in the game addiction scale for adolescents. Cyberpsychol Behav Soc Netw.

[6] Charlton JP, Danforth IDW (2007). Distinguishing addiction and high engagement in the context of online game playing. Comput Hum Behav.

[7] Schivinski B, Brzozowska-Woś M, Buchanan EM, Griffiths MD, Pontes HM (2018). Psychometric assessment of the internet gaming disorder diagnostic criteria: An item response theory study. Addict Behav Rep.

[8] Kim HS, Son G, Roh EB, Ahn WY, Kim J, Shin SH (2022). Prevalence of gaming disorder: A meta-analysis. Addict Behav.

[9] Gao Y-X, Wang J-Y, Dong G-H (2022). The prevalence and possible risk factors of internet gaming disorder among adolescents and young adults: Systematic reviews and meta-analyses. J Psychiatr Res.

[10] Mihara S, Higuchi S (2017). Cross-sectional and longitudinal epidemiological studies of internet gaming disorder: A systematic review of the literature. Psychiatry Clin Neurosci.

[11] Norwegian Media Authority. Barn og Medier 2020. Norwegian Media Authority; 2020.

[12] Chen KH, Oliffe JL, Kelly MT. Internet gaming disorder: An emergent health issue for men. Am J Mens Health. 2018;12(4):1151–9. 10.1177/155798831876695. 10.1177/1557988318766950 PMC6131461 29606034

[13] Lopez-Fernandez O, Williams AJ, Griffiths MD, Kuss DJ (2019). Female gaming, gaming addiction, and the role of women within gaming culture: A narrative literature review. Frontiers in Psychiatry.

[14] González-Bueso V, Santamaría JJ, Fernández D, Merino L, Montero E, Ribas J (2018). Association between internet gaming disorder or pathological video-game use and comorbid psychopathology: A comprehensive review. Int J Environ Res Public Health..

[15] Männikkö N, Ruotsalainen H, Miettunen J, Pontes HM, Kääriäinen M (2017). Problematic gaming behaviour and health-related outcomes: A systematic review and meta-analysis. J Health Psychol.

[16] Kristensen JH, Pallesen S, King DL, Hysing M, Erevik EK (2021). Problematic gaming and sleep: A systematic review and meta-analysis. Front Psychiatry.

[17] Erevik EK, Landrø H, Mattson ÅL, Kristensen JH, Kaur P, Pallesen S (2022). Problem gaming and suicidality: A systematic literature review. Addictive Behaviors Reports.

[18] Junus A, Hsu Y-c, Wong C, Yip PSF (2023). Is internet gaming disorder associated with suicidal behaviors among the younger generation? Multiple logistic regressions on a large-scale purposive sampling survey. J Psychiatr Res.

[19] Ohayon MM, Roberts L (2021). Internet gaming disorder and comorbidities among campus-dwelling U.S. university students. Psychiatry Res.

[20] Evren C, Evren B, Dalbudak E, Topcu M, Kutlu N (2020). Relationship of internet gaming disorder symptom severity with non-suicidal self-injury among young adults. Dusunen Adam The Journal of Psychiatry and Neurological Sciences.

[21] Krossbakken E, Pallesen S, Mentzoni RA, King DL, Molde H, Finserås TR, Torsheim T (2018). A Cross-lagged study of developmental trajectories of video game engagement, addiction, and mental health. Front Psychol.

[22] Hufford MR (2001). Alcohol and suicidal behavior. Clin Psychol Rev.

[23] Khantzian EJ (1997). The self-medication hypothesis of substance use disorders: A reconsideration and recent applications. Harv Rev Psychiatry.

[24] Khantzian EJ (1985). The self-medication hypothesis of addictive disorders: focus on heroin and cocaine dependence. Am J Psychiatry.

[25] Klonsky ED (2007). The functions of deliberate self-injury: a review of the evidence. Clin Psychol Rev.

[26] Myrseth H, Notelaers G, Strand LÅ, Borud EK, Olsen OK (2017). Introduction of a new instrument to measure motivation for gaming: the electronic gaming motives questionnaire. Addiction.

[27] Pan P-Y, Yeh C-B (2018). Internet Addiction among Adolescents May Predict Self-Harm/Suicidal Behavior: A Prospective Study. J Pediatr.

[28] Xiong A, Liao S, Luo B, Luo S, Tong Y, Li Z (2023). Associations between problematic internet use, life satisfaction, and deliberate self-harm among Chinese adolescents: A multi-centered longitudinal study. Addict Behav.

[29] Charlton JP (2002). A factor-analytic investigation of computer ‘addiction’ and engagement. Br J Psychol.

[30] André F, Broman N, Håkansson A, Claesdotter-Knutsson E (2020). Gaming addiction, problematic gaming and engaged gaming – Prevalence and associated characteristics. Addictive Behaviors Reports.

[31] Brunborg GS, Mentzoni RA, Melkevik OR, Torsheim T, Samdal O, Hetland J (2013). Gaming addiction, gaming engagement, and psychological health complaints among norwegian adolescents. Media Psychol.

[32] Ferguson CJ, Coulson M, Barnett J (2011). A meta-analysis of pathological gaming prevalence and comorbidity with mental health, academic and social problems. J Psychiatr Res.

[33] Finserås TR, Sivertsen B, Pallesen S, Leino T, Mentzoni RA, Skogen JC (2022). Different typologies of gamers are associated with mental health: Are students DOOMed?. Int J Environ Res Public Health.

[34] Madge N, Hewitt A, Hawton K, Wilde EJd, Corcoran P, Fekete S (2008). Deliberate self-harm within an international community sample of young people: comparative findings from the Child & Adolescent Self-harm in Europe (CASE) Study. J Child Psychol Psychiatry.

[35] McManus S, Bebbington PE, Jenkins R, Brugha T. Mental health and wellbeing in England: the adult psychiatric morbidity survey 2014. NHS digital.; 2016.

[36] Lemmens JS, Valkenburg PM, Peter J (2009). Development and validation of a game addiction scale for adolescents. Media Psychol.

[37] Wittek CT, Finserås TR, Pallesen S, Mentzoni RA, Hanss D, Griffiths MD, Molde H (2016). Prevalence and predictors of video game addiction: A study based on a national representative sample of gamers. Int J Ment Heal Addict.

[38] Haug E, Mæland S, Lehmann S, Bjørknes R, Fadnes LT, Sandal GM, Skogen JC (2022). Increased gaming during COVID-19 predicts physical inactivity among youth in Norway—A two-wave longitudinal cohort study. Frontiers in Public Health..

[39] Strengthening the reporting of observational studies in epidemiology (STROBE): Explanation and elaboration. Ann Intern Med. 2007;147(8):W-163-W-94. 10.7326/0003-4819-147-8-200710160-00010-w1 17938389

[40] McLean L, Griffiths MD (2019). Female gamers’ experience of online harassment and social support in online gaming: A aualitative study. Int J Ment Heal Addict.

[41] Dong G-H, Potenza MN (2022). Considering gender differences in the study and treatment of internet gaming disorder. J Psychiatr Res.

[42] Melodia F, Canale N, Griffiths MD (2022). The role of avoidance coping and escape motives in problematic online gaming: A systematic literature review. Int J Ment Heal Addict.

[43] Király O, Urbán R, Griffiths MD, Ágoston C, Nagygyörgy K, Kökönyei G, Demetrovics Z (2015). The mediating effect of gaming motivation between psychiatric symptoms and problematic online gaming: an online survey. J Med Internet Res.

[44] Holdsworth L, Hing N, Breen H (2012). Exploring women's problem gambling: a review of the literature. Int Gambl Stud.

[45] Slack JD, Delfabbro P, King DL (2022). Toward a delineation of the differences between high engagement and problem gaming. Addictive Behaviors Reports.

[46] Stedman RC, Connelly NA, Heberlein TA, Decker DJ, Allred SB (2019). The End of the (Research) world as we know it? Understanding and coping with declining response rates to mail surveys. Soc Nat Resour.

[47] Rindfuss RR, Bumpass LL, Choe MK, Tsuya NO, Tamaki E. Do low survey response rates bias results? Evidence from Japan. Demographic Res. 2015;32(26):797–828.

[48] Krumpal I (2013). Determinants of social desirability bias in sensitive surveys: a literature review. Qual Quant.

[49] Coughlin SS (1990). Recall bias in epidemiologic studies. J Clin Epidemiol.

[50] Podsakoff PM, MacKenzie SB, Lee J-Y, Podsakoff NP (2003). Common method biases in behavioral research: A critical review of the literature and recommended remedies. J Appl Psychol.

