# Supplementary material for: Examining the relationship between non-suicidal self-harm and suicidality within the past 12-months and gaming problems in Norwegian full-time students
Source: BMC Psychiatry. 2024 Mar 28;24:234. doi: 10.1186/s12888-024-05694-3 (PMC10976819; doi:10.1186/s12888-024-05694-3)
Supplement: Supplementary file 1 — Supplementary Material 1. [file 12888_2024_5694_MOESM1_ESM.docx]

**
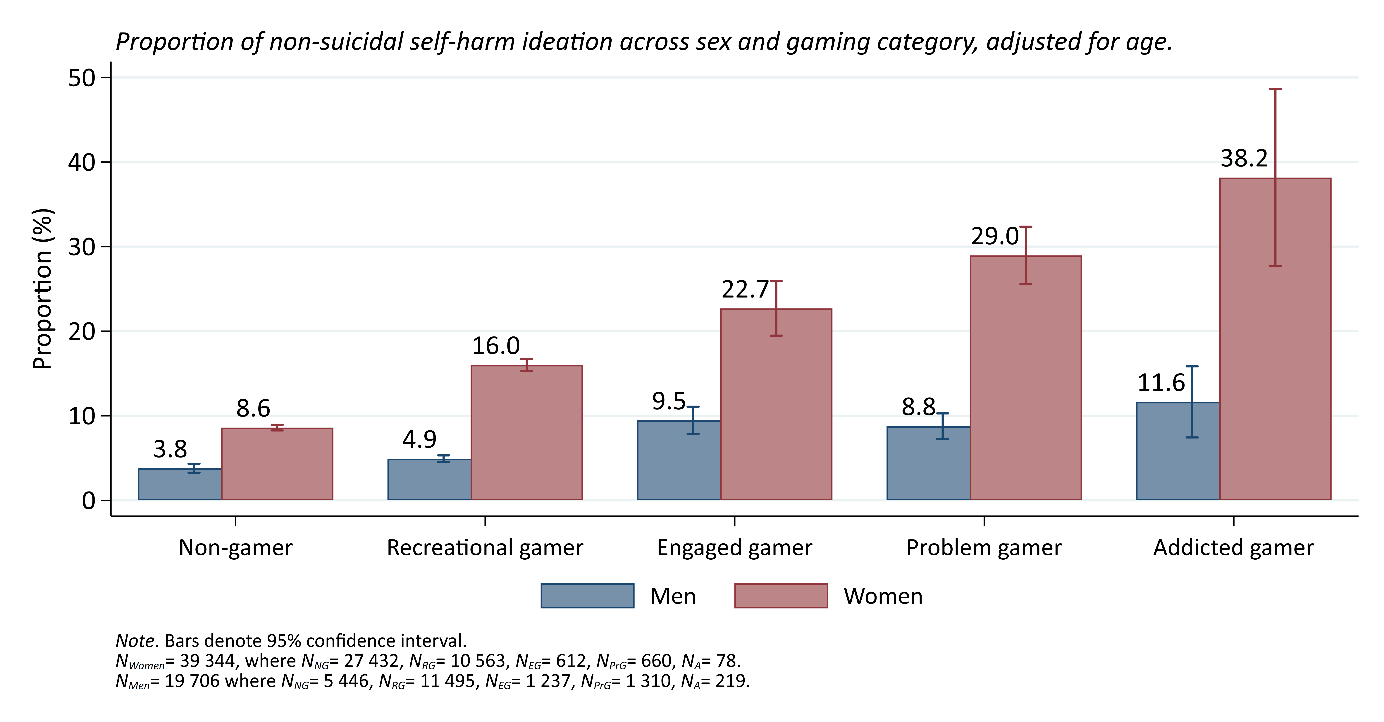
 Figure 1. The proportion of non-suicidal self-harm ideation by sex and gaming category, adjusted for age.**


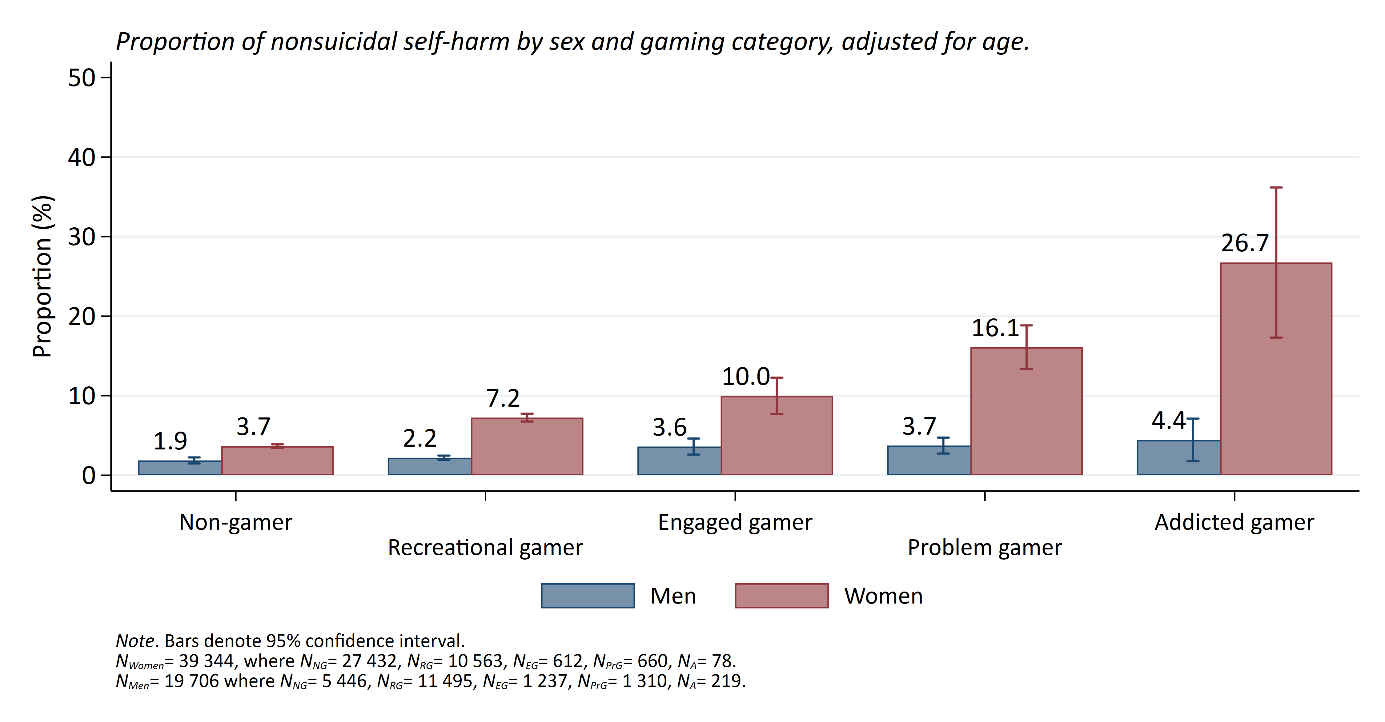
 **Figure 2. The proportion of non-suicidal self-harm by sex and gaming category, adjusted for age.**


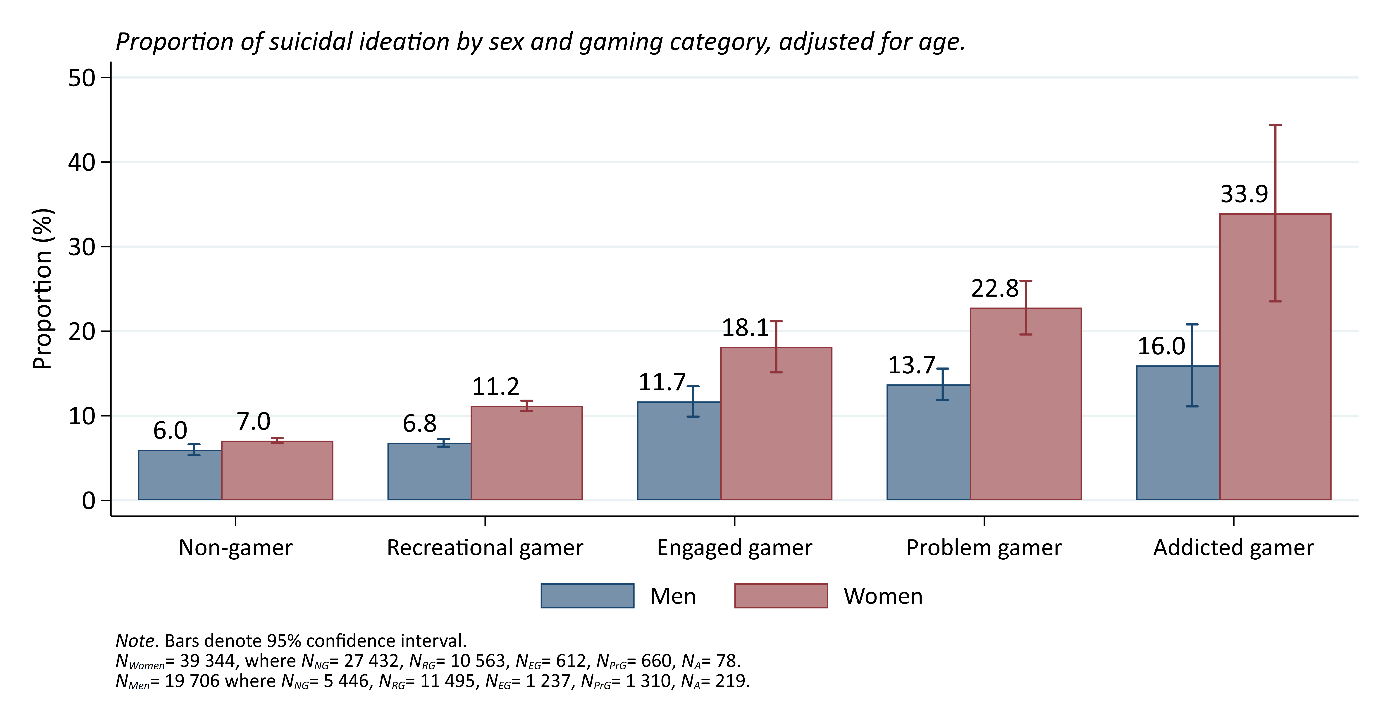


**Figure 3. The proportion of suicidal ideation by sex and gaming category, adjusted for age.**


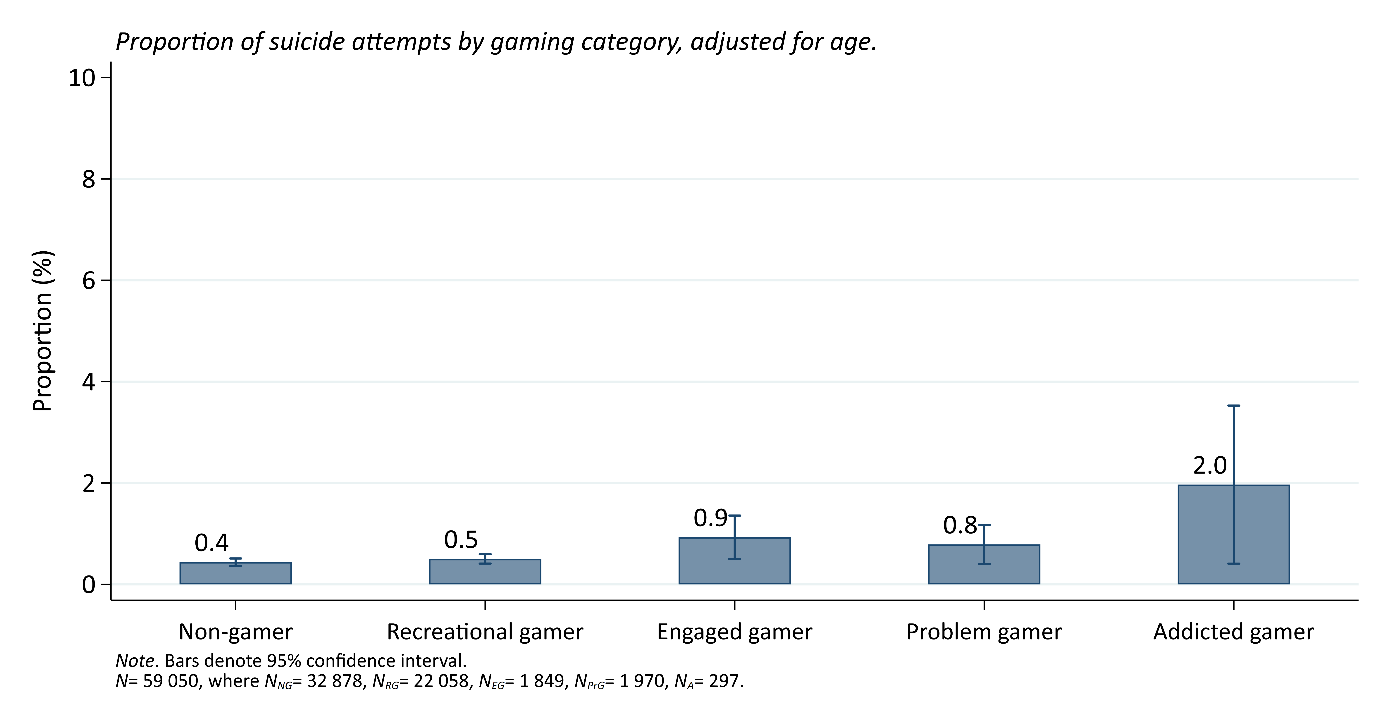
 **Figure 4. show the proportion of suicidal attempt by gaming category, adjusted for age**.
